# 1,3-Bis(2-ethoxy­phen­yl)triazene

**DOI:** 10.1107/S1600536809008034

**Published:** 2009-03-11

**Authors:** Mohammad Kazem Rofouei, Mohammad Reza Melardi, Yasaman Salemi, Saba Razi Kazemi

**Affiliations:** a Faculty of Chemistry, Tarbiat Moallem University, Tehran, Iran; b Department of Chemistry, Islamic Azad University, Karaj Branch, Karaj, Iran

## Abstract

The title compound, C_16_H_19_N_3_O_2_, exhibits a *trans* geometry about the N=N double bond in the triazene unit in the solid state, and individual mol­ecules are close to planar with r.m.s. deviations from planarity of 0.065 Å and 0.242 Å for the two independent molecules in the asymmetric unit. Distinct inter­molecular N—H⋯N hydrogen bonds lead to the formation of dimers with an *R*_2_^2^(8) graph-set motif. The steric demands of the eth­oxy groups in the *ortho* position prevent a coplanar arrangement of the two mol­ecules in the dimers and these instead consist of two inter­locked mol­ecules that are related by a non-crystallographic pseudo-twofold rotation axis. Weak C—H⋯π inter­actions between the CH groups and the aromatic phenyl rings also occur.

## Related literature

For aryl triazenes, their structural properties and metal complexes, see: Meldola *et al.* (1888 ▶); Leman *et al.* (1993 ▶); Chen *et al.* (2002 ▶); Vrieze *et al.* (1987 ▶). For a similar structure with cyano instead of eth­oxy groups, see: Melardi *et al.* (2008 ▶). For the synthesis and characterization of a similar structure with meth­oxy instead of eth­oxy groups, see: Rofouei *et al.* (2006 ▶). For the synthesis and crystal structures of mercury(II) and silver(I) complexes with 1,3-bis­(2-methoxy­phen­yl)tri­azene, see: Hematyar *et al.* (2008 ▶) and Payehghadr *et al.* (2007 ▶), respectively. For the investigation of hydrogen-bond patterns and related graph sets, see: Grell *et al.* (2002 ▶).
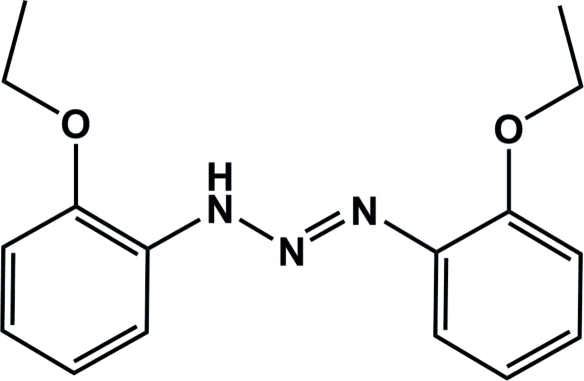


## Experimental

### 

#### Crystal data

C_16_H_19_N_3_O_2_
*M* *_r_* = 285.34
Triclinic, 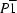

*a* = 11.3971 (7) Å
*b* = 11.8696 (7) Å
*c* = 14.0627 (9) Å
α = 106.467 (5)°
β = 98.598 (5)°
γ = 116.512 (5)°
*V* = 1545.7 (2) Å^3^
*Z* = 4
Mo *K*α radiation
μ = 0.08 mm^−1^
*T* = 120 K
0.30 × 0.20 × 0.15 mm

#### Data collection

Bruker SMART 1000 CCD area-detector diffractometer
Absorption correction: multi-scan (*SADABS*; Bruker, 1998 ▶) *T*_min_ = 0.973, *T*_max_ = 0.982
17109 measured reflections
8181 independent reflections
4988 reflections with *I* > 2σ(*I*)
*R*_int_ = 0.031

#### Refinement

*R*[*F* ^2^ > 2σ(*F* ^2^)] = 0.058
*wR*(*F* ^2^) = 0.138
*S* = 1.00
8181 reflections
383 parameters
H-atom parameters constrained
Δρ_max_ = 0.34 e Å^−3^
Δρ_min_ = −0.31 e Å^−3^

### 

Data collection: *SMART* (Bruker, 1998 ▶); cell refinement: *SAINT-Plus* (Bruker, 1998 ▶); data reduction: *SAINT-Plus*; program(s) used to solve structure: *SHELXTL* (Sheldrick, 2008 ▶); program(s) used to refine structure: *SHELXTL*; molecular graphics: *SHELXTL*; software used to prepare material for publication: *SHELXTL*.

## Supplementary Material

Crystal structure: contains datablocks I, global. DOI: 10.1107/S1600536809008034/zl2178sup1.cif

Structure factors: contains datablocks I. DOI: 10.1107/S1600536809008034/zl2178Isup2.hkl

Additional supplementary materials:  crystallographic information; 3D view; checkCIF report

## Figures and Tables

**Table 1:** Hydrogen-bond geometry (Å, °)

*D*—H⋯*A*	*D*—H	H⋯*A*	*D*⋯*A*	*D*—H⋯*A*
N1—H1*N*⋯N4	0.91	2.12	3.018 (2)	170
N6—H6*N*⋯N3	0.91	2.11	3.008 (2)	170
C4—H4*A*⋯*Cg*1^i^	0.95	2.85	3.686 (2)	147
C32—H32*B*⋯*Cg*2^ii^	0.98	2.78	3.549 (3)	136

Symmetry codes: (i) 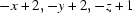; (ii) 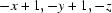. *Cg*1 and *Cg*2 are the centroids of the C17–C22 and C25–C30 rings, respectively.

**Fig. 1.**
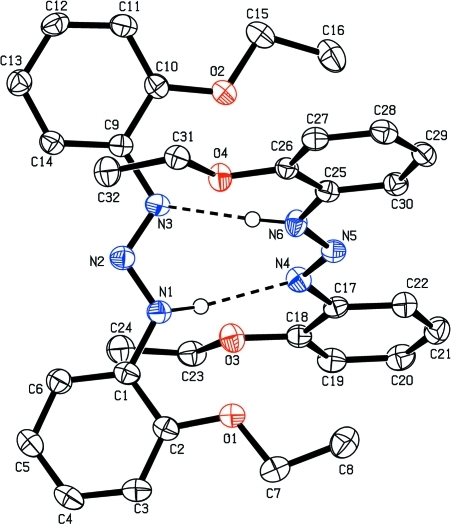
Molecular structure of the title compound. Only hydrogen atoms involved in the hydrogen bonds are shown. Thermal ellipsoids are drawn at the 50% probability level.

**Fig. 2.**
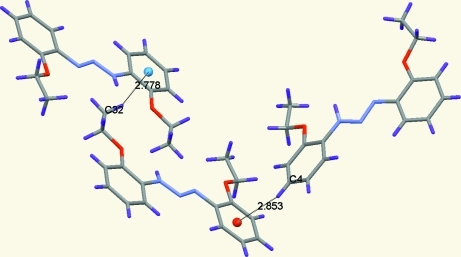
C–H···π Interactions between CH groups with aromatic phenyl rings with H···π distances of 2.85 Å for C4–H4A···Cg1 (2 - x, 2 - y, 1 - z) and 2.78 Å for C32–H32B···Cg2 (1 - x, 1 - y, -z) [Cg1 and Cg2 are centroids for C17—C22 and C25—C30 rings, respectively].

**Fig. 3.**
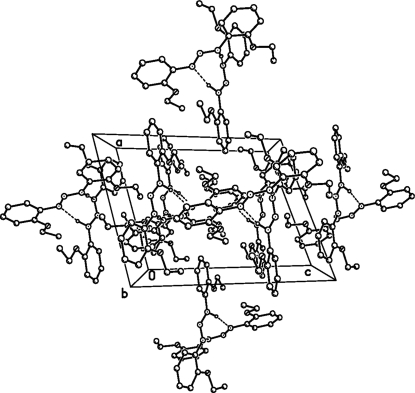
Unit cell packing diagram of the title compound. Hydrogen bonds are shown as dashed lines.

## supplementary crystallographic information

### Comment

Aryl triazenes have been studied for over 130 years for their interesting structural, anticancer, and reactivity properties. The first extensive investigation of the coordination chemistry of a triazene derivative (1,3-diphenyltriazene) was carried out in 1887 by Meldola (Meldola *et al.*, 1888). In the intervening years, numerous transition metal triazenide compounds have been studied (Leman *et al.*, 1993). Triazene compounds characterized by having a diazoamino group commonly adopt a *trans* configuration in the ground state (Chen *et al.*, 2002). The study of transition metal complexes containing 1,3-diaryltriazenide [RN═N—NR]^-^ ligands has increased greatly in the past few years, because of their potential reactivity in relation to their several coordination modes (Vrieze *et al.*, 1987). We have recently reported the synthesis and characterization of the two molecules 1,3-bis(2-methoxyphenyl)triazene (Rofouei, *et al.*, 2006) and 1,3-bis(2-cyanophenyl)triazene (Melardi, *et al.*, 2008).

The title compound, C_16_H_19_N_3_O_2_, is a related triazene compound and crystallizes in the space group *P*1 with two crystallographically independent molecules per unit cell. It exhibits a *trans* stereo chemistry of the N═N double bond, and the C9—N3—N2—N1 and C17—N4—N5—N6 torsion angles are -179.45 (13) and 176.67 (13)°, respectively. The N1—N2, N2—N3, N4—N5 and N5—N6 bond distances are 1.3196 (18), 1.2909 (18), 1.2896 (18) and 1.3214 (18) Å, respectively, which indicates the presence of distinct single and double bonds between the nitrogen atoms. These values are in good agreement with the reported data for N—N and N═N bond distances (Hematyar, *et al.*, 2008; Payehghadr, *et al.* 2007). For example, in 1,3-bis(2-cyanophenyl)triazene, the N—N and N═N bond distances are 1.335 (5) and 1.289 (5) Å (Melardi, *et al.*, 2008). Individual molecules are mostly planar with an rms deviation from planarity of 0.0646 Å for all non-hydrogen atoms.

The two crystallographically independent molecules in the molecular structure (Fig. 1) are connected by two distinct classic N—H···N hydrogen bonds with D···A distances of 3.018 (2) and 3.008 (2) Å (Table 1). The N—H···N hydrogen bonds lead to the formation of dimers with an *R*_2_^2^(8) graph set geometry (Grell *et al.*, 2002). The steric demand of the ethoxy groups in the ortho position prevents a co-planar arrangement of the two molecules in the dimers and these do instead consist of two interlocked molecules that are related by a non-crystallographic pseudo-twofold rotation axis. The dihedral angle between the best least square planes of the two molecules is 63.15 (3) °.

Also, there are interesting weak C—H···π interactions between the CH groups and the aromatic phenyl rings with H···π and C···π distances of 2.85 and 3.686 (2) Å for C4–H4A···*Cg*1 (2 - *x*, 2 - *y*, 1 - *z*) and 2.78 and 3.549 (3) Å for C32–H32B···*Cg*2 (1 - *x*, 1 - *y*, -*z*) [*Cg*1 and *Cg*2 are centroids for C17—C22 and C25—C30 rings, respectively] (Fig. 2). The unit cell packing of the title compound is presented in Fig. 3.

### Experimental

The compound was prepared by the following method: A 100 ml flask was charged with 10 g of ice and 15 ml of water and then cooled to 273 K in an ice-bath. To this was added 10 mmol (1.37 g) of *o*–phenetidin and 13 mmol of hydrochloric acid (37%). To this solution was added a solution containing NaNO_2_ (6 mmol, 0.41 g) in 25 ml of water during a 15 min period. After mixing for 15 min, a solution containing 180 mmol (14.76 g) of sodium acetate in 45 ml of water was added. After mixing for 45 min the brown product was filtered off and dissolved in Et_2_O, and was crystallized at 263 K. Yield, (50%) 24 mmol (6.85 g). Recrystallization from Et_2_O afforded the product as an orange crystalline material. m. p. 374–375 K. ^1^H NMR(300 MHz, DMSO): 1.36 (6*H*, CH_3_), 4.10 (4*H*, CH_2_), 6.91–7.53 (8*H*, aromatic), 11.26 (1*H*, NH). IR (KBr): 3149, 2977, 1599, 1489, 1253, 1045, 742 cm^-1^.

### Refinement

The hydrogen atoms of the NH groups were found in difference density Fourier maps, but eventually all H atoms were placed in calculated positions. All hydrogen atoms were refined in isotropic approximation using a riding model with the *U*_iso_(H) parameters equal to 1.2 *U*_eq_(C/N), for methyl groups equal to 1.5 *U*_eq_(C), where U(C) and U(N) are the respective equivalent thermal parameters of the carbon and nitrogen atoms to which the corresponding H atoms are bonded. The C-H distances are in the range of 0.95–0.98 Å, N-H distances are 0.91 Å.

### Crystal data

**Table:**

C_16_H_19_N_3_O_2_	*Z* = 4
*M**_r_* = 285.34	*F*(000) = 608
Triclinic, *P*1	*D*_x_ = 1.226 Mg m^−^^3^
Hall symbol: -P 1	Mo *K*α radiation, λ = 0.71073 Å
*a* = 11.3971 (7) Å	Cell parameters from 887 reflections
*b* = 11.8696 (7) Å	θ = 3–30°
*c* = 14.0627 (9) Å	µ = 0.08 mm^−^^1^
α = 106.467 (5)°	*T* = 120 K
β = 98.598 (5)°	Prism, orange
γ = 116.512 (5)°	0.30 × 0.20 × 0.15 mm
*V* = 1545.7 (2) Å^3^	

### Data collection

**Table:**

Bruker SMART 1000 CCD area-detector diffractometer	8181 independent reflections
Radiation source: fine-focus sealed tube	4988 reflections with *I* > 2σ(*I*)
graphite	*R*_int_ = 0.031
φ and ω scans	θ_max_ = 29.0°, θ_min_ = 1.6°
Absorption correction: multi-scan (*SADABS*; Bruker, 1998)	*h* = −15→15
*T*_min_ = 0.973, *T*_max_ = 0.982	*k* = −16→16
17109 measured reflections	*l* = −19→19

### Refinement

**Table:**

Refinement on *F*^2^	Primary atom site location: structure-invariant direct methods
Least-squares matrix: full	Secondary atom site location: difference Fourier map
*R*[*F*^2^ > 2σ(*F*^2^)] = 0.058	Hydrogen site location: mixed
*wR*(*F*^2^) = 0.138	H-atom parameters constrained
*S* = 1.00	*w* = 1/[σ^2^(*F*_o_^2^) + (0.0517*P*)^2^ + 0.497*P*] where *P* = (*F*_o_^2^ + 2*F*_c_^2^)/3
8181 reflections	(Δ/σ)_max_ < 0.001
383 parameters	Δρ_max_ = 0.34 e Å^−^^3^
0 restraints	Δρ_min_ = −0.31 e Å^−^^3^

### Special details

**Table:**

Geometry. All e.s.d.'s (except the e.s.d. in the dihedral angle between two l.s. planes) are estimated using the full covariance matrix. The cell e.s.d.'s are taken into account individually in the estimation of e.s.d.'s in distances, angles and torsion angles; correlations between e.s.d.'s in cell parameters are only used when they are defined by crystal symmetry. An approximate (isotropic) treatment of cell e.s.d.'s is used for estimating e.s.d.'s involving l.s. planes.
Refinement. Refinement of *F*^2^ against ALL reflections. The weighted *R*-factor *wR* and goodness of fit *S* are based on *F*^2^, conventional *R*-factors *R* are based on *F*, with *F* set to zero for negative *F*^2^. The threshold expression of *F*^2^ > σ(*F*^2^) is used only for calculating *R*-factors(gt) *etc*. and is not relevant to the choice of reflections for refinement. *R*-factors based on *F*^2^ are statistically about twice as large as those based on *F*, and *R*- factors based on ALL data will be even larger.

### Fractional atomic coordinates and isotropic or equivalent isotropic displacement parameters (Å^2^)

**Table:**

	*x*	*y*	*z*	*U*_iso_*/*U*_eq_	
N1	0.65754 (14)	0.89887 (15)	0.26280 (11)	0.0273 (3)	
H1N	0.6187	0.8521	0.3017	0.033*	
N2	0.57346 (14)	0.91539 (14)	0.20038 (10)	0.0247 (3)	
N3	0.44508 (14)	0.83193 (14)	0.18591 (11)	0.0253 (3)	
O1	0.81959 (12)	0.82358 (12)	0.34265 (9)	0.0309 (3)	
O2	0.17841 (12)	0.66700 (12)	0.14955 (9)	0.0308 (3)	
C1	0.80038 (16)	0.98331 (17)	0.28546 (12)	0.0244 (4)	
C2	0.88658 (17)	0.94198 (17)	0.32698 (13)	0.0256 (4)	
C3	1.02882 (17)	1.01982 (18)	0.34861 (14)	0.0304 (4)	
H3A	1.0873	0.9918	0.3765	0.036*	
C4	1.08542 (18)	1.13829 (18)	0.32955 (14)	0.0323 (4)	
H4A	1.1827	1.1913	0.3445	0.039*	
C5	1.00087 (18)	1.17975 (18)	0.28886 (14)	0.0317 (4)	
H5A	1.0401	1.2610	0.2758	0.038*	
C6	0.85879 (17)	1.10262 (17)	0.26714 (13)	0.0277 (4)	
H6A	0.8010	1.1315	0.2396	0.033*	
C7	0.90069 (18)	0.77429 (18)	0.38370 (14)	0.0314 (4)	
H7A	0.9703	0.8444	0.4523	0.038*	
H7B	0.9498	0.7535	0.3353	0.038*	
C8	0.8034 (2)	0.6466 (2)	0.39582 (15)	0.0386 (5)	
H8A	0.8561	0.6107	0.4247	0.058*	
H8B	0.7358	0.5775	0.3273	0.058*	
H8C	0.7548	0.6682	0.4434	0.058*	
C9	0.35033 (16)	0.84446 (16)	0.11871 (12)	0.0217 (3)	
C10	0.20907 (17)	0.75634 (16)	0.10018 (13)	0.0244 (3)	
C11	0.10996 (17)	0.76308 (17)	0.03477 (13)	0.0278 (4)	
H11A	0.0143	0.7043	0.0226	0.033*	
C12	0.15150 (18)	0.85612 (18)	−0.01262 (14)	0.0294 (4)	
H12A	0.0837	0.8594	−0.0582	0.035*	
C13	0.29049 (18)	0.94394 (17)	0.00583 (13)	0.0279 (4)	
H13A	0.3180	1.0074	−0.0268	0.034*	
C14	0.38903 (17)	0.93889 (17)	0.07190 (12)	0.0245 (3)	
H14A	0.4845	1.0006	0.0857	0.029*	
C15	0.03901 (18)	0.59545 (18)	0.14986 (14)	0.0310 (4)	
H15A	−0.0245	0.5332	0.0776	0.037*	
H15B	0.0101	0.6613	0.1796	0.037*	
C16	0.0351 (2)	0.5146 (2)	0.21591 (15)	0.0396 (5)	
H16A	−0.0596	0.4627	0.2168	0.059*	
H16B	0.0970	0.5775	0.2876	0.059*	
H16C	0.0656	0.4511	0.1864	0.059*	
N4	0.50110 (14)	0.71697 (14)	0.36671 (10)	0.0249 (3)	
N5	0.42582 (13)	0.58882 (14)	0.30543 (10)	0.0230 (3)	
N6	0.39770 (14)	0.57030 (13)	0.20569 (10)	0.0246 (3)	
H6N	0.4166	0.6468	0.1933	0.030*	
O3	0.65249 (12)	0.97589 (11)	0.49894 (9)	0.0283 (3)	
O4	0.36188 (12)	0.53622 (11)	0.00756 (8)	0.0262 (3)	
C17	0.52863 (16)	0.74425 (16)	0.47504 (12)	0.0220 (3)	
C18	0.60555 (16)	0.88364 (17)	0.54400 (13)	0.0238 (3)	
C19	0.62867 (17)	0.91875 (18)	0.65110 (13)	0.0277 (4)	
H19A	0.6786	1.0125	0.6978	0.033*	
C20	0.57898 (18)	0.81711 (19)	0.68985 (13)	0.0305 (4)	
H20A	0.5950	0.8417	0.7630	0.037*	
C21	0.50605 (18)	0.67988 (19)	0.62246 (13)	0.0303 (4)	
H21A	0.4743	0.6106	0.6494	0.036*	
C22	0.47978 (17)	0.64436 (17)	0.51545 (13)	0.0261 (4)	
H22A	0.4276	0.5503	0.4692	0.031*	
C23	0.70481 (18)	1.11735 (17)	0.56228 (13)	0.0290 (4)	
H23A	0.7870	1.1528	0.6224	0.035*	
H23B	0.6332	1.1272	0.5895	0.035*	
C24	0.74328 (19)	1.19540 (18)	0.49298 (14)	0.0328 (4)	
H24A	0.7725	1.2913	0.5318	0.049*	
H24B	0.6629	1.1546	0.4309	0.049*	
H24C	0.8194	1.1910	0.4713	0.049*	
C25	0.31797 (16)	0.43556 (16)	0.12976 (12)	0.0217 (3)	
C26	0.30226 (16)	0.41821 (16)	0.02469 (12)	0.0222 (3)	
C27	0.22978 (17)	0.28660 (17)	−0.05366 (13)	0.0251 (4)	
H27A	0.2193	0.2742	−0.1248	0.030*	
C28	0.17266 (17)	0.17315 (17)	−0.02762 (13)	0.0274 (4)	
H28A	0.1229	0.0834	−0.0813	0.033*	
C29	0.18774 (17)	0.19007 (17)	0.07571 (13)	0.0269 (4)	
H29A	0.1484	0.1122	0.0929	0.032*	
C30	0.26063 (16)	0.32121 (17)	0.15456 (13)	0.0243 (3)	
H30A	0.2713	0.3328	0.2256	0.029*	
C31	0.35336 (17)	0.52266 (17)	−0.09816 (12)	0.0250 (4)	
H31A	0.2552	0.4705	−0.1429	0.030*	
H31B	0.3990	0.4729	−0.1264	0.030*	
C32	0.42454 (19)	0.66412 (18)	−0.09738 (14)	0.0319 (4)	
H32A	0.4228	0.6579	−0.1686	0.048*	
H32B	0.5208	0.7156	−0.0514	0.048*	
H32C	0.3765	0.7112	−0.0716	0.048*	

### Atomic displacement parameters (Å^2^)

**Table:**

	*U*^11^	*U*^22^	*U*^33^	*U*^12^	*U*^13^	*U*^23^
N1	0.0217 (7)	0.0325 (8)	0.0272 (7)	0.0116 (6)	0.0069 (6)	0.0158 (6)
N2	0.0240 (7)	0.0241 (7)	0.0238 (7)	0.0121 (6)	0.0067 (6)	0.0079 (6)
N3	0.0201 (7)	0.0265 (7)	0.0267 (7)	0.0104 (6)	0.0061 (6)	0.0106 (6)
O1	0.0236 (6)	0.0315 (7)	0.0372 (7)	0.0129 (5)	0.0067 (5)	0.0170 (6)
O2	0.0227 (6)	0.0320 (7)	0.0383 (7)	0.0114 (5)	0.0107 (5)	0.0189 (6)
C1	0.0208 (8)	0.0277 (9)	0.0197 (8)	0.0104 (7)	0.0064 (6)	0.0066 (7)
C2	0.0225 (8)	0.0259 (9)	0.0239 (8)	0.0106 (7)	0.0073 (7)	0.0073 (7)
C3	0.0232 (9)	0.0350 (10)	0.0314 (9)	0.0156 (8)	0.0068 (7)	0.0109 (8)
C4	0.0202 (8)	0.0325 (10)	0.0338 (10)	0.0071 (8)	0.0093 (7)	0.0098 (8)
C5	0.0274 (9)	0.0282 (9)	0.0328 (10)	0.0090 (8)	0.0112 (8)	0.0116 (8)
C6	0.0255 (9)	0.0286 (9)	0.0277 (9)	0.0128 (8)	0.0085 (7)	0.0112 (7)
C7	0.0317 (10)	0.0348 (10)	0.0280 (9)	0.0203 (8)	0.0061 (8)	0.0095 (8)
C8	0.0453 (12)	0.0378 (11)	0.0335 (10)	0.0230 (10)	0.0090 (9)	0.0147 (9)
C9	0.0214 (8)	0.0211 (8)	0.0214 (8)	0.0116 (7)	0.0063 (6)	0.0059 (6)
C10	0.0241 (8)	0.0226 (8)	0.0260 (8)	0.0116 (7)	0.0100 (7)	0.0086 (7)
C11	0.0212 (8)	0.0252 (9)	0.0325 (9)	0.0112 (7)	0.0062 (7)	0.0080 (7)
C12	0.0273 (9)	0.0314 (10)	0.0327 (9)	0.0193 (8)	0.0057 (7)	0.0118 (8)
C13	0.0297 (9)	0.0267 (9)	0.0303 (9)	0.0162 (8)	0.0100 (7)	0.0121 (7)
C14	0.0219 (8)	0.0234 (8)	0.0270 (9)	0.0112 (7)	0.0087 (7)	0.0087 (7)
C15	0.0246 (9)	0.0292 (9)	0.0327 (10)	0.0101 (8)	0.0117 (7)	0.0088 (8)
C16	0.0380 (11)	0.0346 (11)	0.0339 (10)	0.0085 (9)	0.0141 (9)	0.0132 (9)
N4	0.0262 (7)	0.0235 (7)	0.0200 (7)	0.0110 (6)	0.0065 (6)	0.0056 (6)
N5	0.0222 (7)	0.0240 (7)	0.0226 (7)	0.0124 (6)	0.0073 (6)	0.0080 (6)
N6	0.0311 (8)	0.0194 (7)	0.0207 (7)	0.0117 (6)	0.0068 (6)	0.0074 (6)
O3	0.0328 (7)	0.0205 (6)	0.0257 (6)	0.0112 (5)	0.0091 (5)	0.0055 (5)
O4	0.0327 (7)	0.0221 (6)	0.0201 (6)	0.0118 (5)	0.0081 (5)	0.0074 (5)
C17	0.0213 (8)	0.0255 (8)	0.0215 (8)	0.0145 (7)	0.0068 (6)	0.0081 (7)
C18	0.0220 (8)	0.0262 (9)	0.0252 (8)	0.0139 (7)	0.0089 (7)	0.0095 (7)
C19	0.0259 (9)	0.0292 (9)	0.0231 (8)	0.0140 (8)	0.0062 (7)	0.0049 (7)
C20	0.0320 (10)	0.0398 (11)	0.0214 (8)	0.0206 (9)	0.0089 (7)	0.0108 (8)
C21	0.0332 (10)	0.0340 (10)	0.0288 (9)	0.0190 (8)	0.0120 (8)	0.0155 (8)
C22	0.0274 (9)	0.0252 (9)	0.0248 (8)	0.0137 (7)	0.0084 (7)	0.0085 (7)
C23	0.0275 (9)	0.0235 (9)	0.0289 (9)	0.0131 (7)	0.0046 (7)	0.0034 (7)
C24	0.0338 (10)	0.0253 (9)	0.0356 (10)	0.0151 (8)	0.0087 (8)	0.0088 (8)
C25	0.0196 (8)	0.0198 (8)	0.0234 (8)	0.0105 (7)	0.0057 (6)	0.0055 (6)
C26	0.0192 (8)	0.0208 (8)	0.0255 (8)	0.0102 (7)	0.0065 (6)	0.0082 (7)
C27	0.0240 (8)	0.0261 (9)	0.0217 (8)	0.0128 (7)	0.0055 (7)	0.0059 (7)
C28	0.0239 (9)	0.0205 (8)	0.0301 (9)	0.0105 (7)	0.0056 (7)	0.0031 (7)
C29	0.0221 (8)	0.0215 (8)	0.0351 (10)	0.0097 (7)	0.0101 (7)	0.0106 (7)
C30	0.0229 (8)	0.0277 (9)	0.0237 (8)	0.0135 (7)	0.0089 (7)	0.0104 (7)
C31	0.0245 (8)	0.0277 (9)	0.0203 (8)	0.0124 (7)	0.0077 (7)	0.0078 (7)
C32	0.0373 (10)	0.0334 (10)	0.0275 (9)	0.0181 (8)	0.0142 (8)	0.0133 (8)

### Geometric parameters (Å, °)

**Table:**

N1—N2	1.3196 (18)	N4—N5	1.2896 (18)
N1—C1	1.401 (2)	N4—C17	1.418 (2)
N1—H1N	0.9100	N5—N6	1.3214 (18)
N2—N3	1.2909 (18)	N6—C25	1.403 (2)
N3—C9	1.414 (2)	N6—H6N	0.9100
O1—C2	1.369 (2)	O3—C18	1.3634 (19)
O1—C7	1.430 (2)	O3—C23	1.4380 (19)
O2—C10	1.3739 (19)	O4—C26	1.3684 (19)
O2—C15	1.430 (2)	O4—C31	1.4327 (18)
C1—C6	1.390 (2)	C17—C22	1.388 (2)
C1—C2	1.403 (2)	C17—C18	1.410 (2)
C2—C3	1.389 (2)	C18—C19	1.392 (2)
C3—C4	1.386 (2)	C19—C20	1.389 (2)
C3—H3A	0.9500	C19—H19A	0.9500
C4—C5	1.384 (3)	C20—C21	1.387 (2)
C4—H4A	0.9500	C20—H20A	0.9500
C5—C6	1.387 (2)	C21—C22	1.387 (2)
C5—H5A	0.9500	C21—H21A	0.9500
C6—H6A	0.9500	C22—H22A	0.9500
C7—C8	1.503 (3)	C23—C24	1.509 (2)
C7—H7A	0.9900	C23—H23A	0.9900
C7—H7B	0.9900	C23—H23B	0.9900
C8—H8A	0.9800	C24—H24A	0.9800
C8—H8B	0.9800	C24—H24B	0.9800
C8—H8C	0.9800	C24—H24C	0.9800
C9—C14	1.396 (2)	C25—C30	1.391 (2)
C9—C10	1.404 (2)	C25—C26	1.405 (2)
C10—C11	1.393 (2)	C26—C27	1.392 (2)
C11—C12	1.390 (2)	C27—C28	1.393 (2)
C11—H11A	0.9500	C27—H27A	0.9500
C12—C13	1.383 (2)	C28—C29	1.382 (2)
C12—H12A	0.9500	C28—H28A	0.9500
C13—C14	1.381 (2)	C29—C30	1.391 (2)
C13—H13A	0.9500	C29—H29A	0.9500
C14—H14A	0.9500	C30—H30A	0.9500
C15—C16	1.505 (3)	C31—C32	1.499 (2)
C15—H15A	0.9900	C31—H31A	0.9900
C15—H15B	0.9900	C31—H31B	0.9900
C16—H16A	0.9800	C32—H32A	0.9800
C16—H16B	0.9800	C32—H32B	0.9800
C16—H16C	0.9800	C32—H32C	0.9800
			
N2—N1—C1	117.93 (14)	N5—N4—C17	114.76 (13)
N2—N1—H1N	115.2	N4—N5—N6	111.94 (13)
C1—N1—H1N	124.3	N5—N6—C25	118.29 (13)
N3—N2—N1	111.87 (13)	N5—N6—H6N	115.3
N2—N3—C9	114.20 (13)	C25—N6—H6N	125.1
C2—O1—C7	118.23 (13)	C18—O3—C23	117.69 (13)
C10—O2—C15	117.52 (13)	C26—O4—C31	117.43 (12)
C6—C1—N1	123.34 (15)	C22—C17—C18	119.30 (15)
C6—C1—C2	119.40 (15)	C22—C17—N4	124.46 (15)
N1—C1—C2	117.25 (15)	C18—C17—N4	116.16 (14)
O1—C2—C3	125.06 (15)	O3—C18—C19	124.44 (15)
O1—C2—C1	115.10 (14)	O3—C18—C17	116.05 (14)
C3—C2—C1	119.84 (15)	C19—C18—C17	119.51 (15)
C4—C3—C2	120.06 (16)	C20—C19—C18	120.20 (16)
C4—C3—H3A	120.0	C20—C19—H19A	119.9
C2—C3—H3A	120.0	C18—C19—H19A	119.9
C5—C4—C3	120.33 (16)	C21—C20—C19	120.42 (16)
C5—C4—H4A	119.8	C21—C20—H20A	119.8
C3—C4—H4A	119.8	C19—C20—H20A	119.8
C4—C5—C6	119.94 (16)	C20—C21—C22	119.60 (16)
C4—C5—H5A	120.0	C20—C21—H21A	120.2
C6—C5—H5A	120.0	C22—C21—H21A	120.2
C5—C6—C1	120.43 (16)	C21—C22—C17	120.93 (16)
C5—C6—H6A	119.8	C21—C22—H22A	119.5
C1—C6—H6A	119.8	C17—C22—H22A	119.5
O1—C7—C8	107.45 (15)	O3—C23—C24	106.93 (14)
O1—C7—H7A	110.2	O3—C23—H23A	110.3
C8—C7—H7A	110.2	C24—C23—H23A	110.3
O1—C7—H7B	110.2	O3—C23—H23B	110.3
C8—C7—H7B	110.2	C24—C23—H23B	110.3
H7A—C7—H7B	108.5	H23A—C23—H23B	108.6
C7—C8—H8A	109.5	C23—C24—H24A	109.5
C7—C8—H8B	109.5	C23—C24—H24B	109.5
H8A—C8—H8B	109.5	H24A—C24—H24B	109.5
C7—C8—H8C	109.5	C23—C24—H24C	109.5
H8A—C8—H8C	109.5	H24A—C24—H24C	109.5
H8B—C8—H8C	109.5	H24B—C24—H24C	109.5
C14—C9—C10	119.11 (14)	C30—C25—N6	123.08 (14)
C14—C9—N3	124.14 (14)	C30—C25—C26	119.77 (14)
C10—C9—N3	116.74 (14)	N6—C25—C26	117.08 (14)
O2—C10—C11	124.14 (15)	O4—C26—C27	124.58 (14)
O2—C10—C9	116.04 (14)	O4—C26—C25	115.80 (14)
C11—C10—C9	119.82 (15)	C27—C26—C25	119.62 (14)
C12—C11—C10	119.79 (15)	C26—C27—C28	119.93 (15)
C12—C11—H11A	120.1	C26—C27—H27A	120.0
C10—C11—H11A	120.1	C28—C27—H27A	120.0
C13—C12—C11	120.73 (16)	C29—C28—C27	120.51 (15)
C13—C12—H12A	119.6	C29—C28—H28A	119.7
C11—C12—H12A	119.6	C27—C28—H28A	119.7
C14—C13—C12	119.60 (16)	C28—C29—C30	119.94 (15)
C14—C13—H13A	120.2	C28—C29—H29A	120.0
C12—C13—H13A	120.2	C30—C29—H29A	120.0
C13—C14—C9	120.92 (15)	C29—C30—C25	120.22 (15)
C13—C14—H14A	119.5	C29—C30—H30A	119.9
C9—C14—H14A	119.5	C25—C30—H30A	119.9
O2—C15—C16	107.22 (15)	O4—C31—C32	107.73 (13)
O2—C15—H15A	110.3	O4—C31—H31A	110.2
C16—C15—H15A	110.3	C32—C31—H31A	110.2
O2—C15—H15B	110.3	O4—C31—H31B	110.2
C16—C15—H15B	110.3	C32—C31—H31B	110.2
H15A—C15—H15B	108.5	H31A—C31—H31B	108.5
C15—C16—H16A	109.5	C31—C32—H32A	109.5
C15—C16—H16B	109.5	C31—C32—H32B	109.5
H16A—C16—H16B	109.5	H32A—C32—H32B	109.5
C15—C16—H16C	109.5	C31—C32—H32C	109.5
H16A—C16—H16C	109.5	H32A—C32—H32C	109.5
H16B—C16—H16C	109.5	H32B—C32—H32C	109.5
			
C1—N1—N2—N3	−179.87 (14)	C17—N4—N5—N6	176.67 (13)
N1—N2—N3—C9	−179.45 (13)	N4—N5—N6—C25	179.15 (13)
N2—N1—C1—C6	15.8 (2)	N5—N4—C17—C22	−0.5 (2)
N2—N1—C1—C2	−163.08 (14)	N5—N4—C17—C18	−177.30 (14)
C7—O1—C2—C3	−0.5 (2)	C23—O3—C18—C19	−13.0 (2)
C7—O1—C2—C1	179.30 (14)	C23—O3—C18—C17	166.92 (14)
C6—C1—C2—O1	179.75 (14)	C22—C17—C18—O3	178.74 (14)
N1—C1—C2—O1	−1.4 (2)	N4—C17—C18—O3	−4.3 (2)
C6—C1—C2—C3	−0.5 (2)	C22—C17—C18—C19	−1.3 (2)
N1—C1—C2—C3	178.44 (15)	N4—C17—C18—C19	175.66 (14)
O1—C2—C3—C4	180.00 (15)	O3—C18—C19—C20	−178.65 (15)
C1—C2—C3—C4	0.2 (3)	C17—C18—C19—C20	1.4 (2)
C2—C3—C4—C5	0.0 (3)	C18—C19—C20—C21	0.0 (3)
C3—C4—C5—C6	0.1 (3)	C19—C20—C21—C22	−1.6 (3)
C4—C5—C6—C1	−0.3 (3)	C20—C21—C22—C17	1.7 (3)
N1—C1—C6—C5	−178.32 (16)	C18—C17—C22—C21	−0.2 (2)
C2—C1—C6—C5	0.5 (2)	N4—C17—C22—C21	−176.97 (16)
C2—O1—C7—C8	178.55 (14)	C18—O3—C23—C24	−177.46 (14)
N2—N3—C9—C14	−0.7 (2)	N5—N6—C25—C30	3.8 (2)
N2—N3—C9—C10	−179.91 (14)	N5—N6—C25—C26	−173.27 (14)
C15—O2—C10—C11	−12.3 (2)	C31—O4—C26—C27	−2.5 (2)
C15—O2—C10—C9	167.79 (14)	C31—O4—C26—C25	177.30 (13)
C14—C9—C10—O2	−179.35 (14)	C30—C25—C26—O4	179.94 (14)
N3—C9—C10—O2	−0.1 (2)	N6—C25—C26—O4	−2.9 (2)
C14—C9—C10—C11	0.8 (2)	C30—C25—C26—C27	−0.2 (2)
N3—C9—C10—C11	179.99 (15)	N6—C25—C26—C27	176.98 (14)
O2—C10—C11—C12	−179.23 (16)	O4—C26—C27—C28	−179.79 (15)
C9—C10—C11—C12	0.6 (2)	C25—C26—C27—C28	0.4 (2)
C10—C11—C12—C13	−1.1 (3)	C26—C27—C28—C29	−0.2 (2)
C11—C12—C13—C14	0.1 (3)	C27—C28—C29—C30	−0.1 (2)
C12—C13—C14—C9	1.3 (3)	C28—C29—C30—C25	0.3 (2)
C10—C9—C14—C13	−1.8 (2)	N6—C25—C30—C29	−177.13 (15)
N3—C9—C14—C13	179.08 (15)	C26—C25—C30—C29	−0.1 (2)
C10—O2—C15—C16	−175.19 (14)	C26—O4—C31—C32	179.72 (13)

### Hydrogen-bond geometry (Å, °)

**Table:**

*D*—H···*A*	*D*—H	H···*A*	*D*···*A*	*D*—H···*A*
N1—H1N···N4	0.91	2.12	3.018 (2)	170
N6—H6N···N3	0.91	2.11	3.008 (2)	170
C4—H4A···Cg1^i^	0.95	2.85	3.686 (2)	147
C32—H32B···Cg2^ii^	0.98	2.78	3.549 (3)	136

Symmetry codes:  (i) −*x*+2, −*y*+2, −*z*+1; (ii) −*x*+1, −*y*+1, −*z*.
